# Lubiprostone ameliorates the cystic fibrosis mouse intestinal phenotype

**DOI:** 10.1186/1471-230X-10-107

**Published:** 2010-09-15

**Authors:** Robert C De Lisle, Racquel Mueller, Eileen Roach

**Affiliations:** 1 Anatomy & Cell Biology, University of Kansas School of Medicine, Kansas City, KS 66160, USA

## Abstract

**Background:**

Cystic fibrosis (CF) is caused by mutations in the *CFTR *gene that impair the function of CFTR, a cAMP-regulated anion channel. In the small intestine loss of CFTR function creates a dehydrated, acidic luminal environment which is believed to cause an accumulation of mucus, a phenotype characteristic of CF. CF mice have small intestinal bacterial overgrowth, an altered innate immune response, and impaired intestinal transit. We investigated whether lubiprostone, which can activate the CLC2 Cl^- ^channel, would improve the intestinal phenotype in CF mice.

**Methods:**

*Cftr^tm1UNC ^*(CF) and wildtype (WT) littermate mice on the C57BL/6J background were used. Lubiprostone (10 μg/kg-day) was administered by gavage for two weeks. Mucus accumulation was estimated from crypt lumen widths in periodic acid-Schiff base, Alcian blue stained sections. Luminal bacterial load was measured by qPCR for the bacterial 16*S *gene. Gastric emptying and small intestinal transit in fasted mice were assessed using gavaged rhodamine dextran. Gene expression was evaluated by Affymetrix Mouse430 2.0 microarray and qRT-PCR.

**Results:**

Crypt width in control CF mice was 700% that of WT mice (*P *< 0.001). Lubiprostone did not affect WT crypt width but, unexpectedly, increased CF crypt width 22% (*P *= 0.001). Lubiprostone increased bacterial load in WT mice to 490% of WT control levels (*P *= 0.008). Conversely, lubiprostone decreased bacterial overgrowth in CF mice by 60% (*P *= 0.005). Lubiprostone increased gastric emptying at 20 min postgavage in both WT (*P *< 0.001) and CF mice (*P *< 0.001). Lubiprostone enhanced small intestinal transit in WT mice (*P *= 0.024) but not in CF mice (*P *= 0.377). Among other innate immune markers, expression of mast cell genes was elevated 4-to 40-fold in the CF intestine as compared to WT, and lubiprostone treatment of CF mice decreased expression to WT control levels.

**Conclusions:**

These results indicate that lubiprostone has some benefits for the CF intestinal phenotype, especially on bacterial overgrowth and the innate immune response. The unexpected observation of increased mucus accumulation in the crypts of lubiprostone-treated CF mice suggests the possibility that lubiprostone increases mucus secretion.

## Background

Cystic fibrosis (CF) is an autosomal recessive disease caused by mutations in the gene encoding the cystic fibrosis transmembrane conductance regulator (CFTR), a cAMP-regulated anion channel. CFTR is crucial for epithelial Cl^-^-dependent fluid transport and bicarbonate ion-dependent pH regulation of luminal spaces. As a consequence, in CF, epithelial surfaces are poorly hydrated and abnormally acidic. These conditions contribute to impaired turnover of the protective mucus layer on epithelial surfaces. Accumulation of mucus is permissive for abnormal microbial colonization and growth which leads to inflammation and tissue damage.

Currently used treatments for CF are largely aimed at symptomatic relief. Various novel approaches that correct mutant CFTR function [1], or activate alternate electrolyte secretory mechanisms are under investigation [2]. These new approaches are attractive therapeutic options as they would correct the underlying defect and hence are expected to also improve the subsequent complications that occur due to loss of CFTR function.

The CF mouse gastrointestinal tract provides a useful model to test potential therapies because of the wide-ranging and dramatic changes that occur compared to control WT mice. These changes also occur in human CF patients and include poor body weight gain [3,4], accumulation of luminal mucus [5,6], microbial colonization of the mucus resulting in bacterial overgrowth [4,5,7], altered innate immune response [8,9], and impaired gastrointestinal motility [10-12]. We have proposed that the initiating event in the intestinal pathogenesis in CF is slowed turnover of luminal mucus due to the decreased fluid secretion and abnormally acidic pH of the luminal fluid that occur as a result of lack of normal CFTR function. The static mucus in the CF mouse small intestine becomes overgrown with colonic-type bacteria which upregulate some innate defenses, including a dramatic influx of mast cells [9] and altered prostaglandin metabolism [13].

We have shown previously that administration of an oral osmotic laxative largely corrects the intestinal phenotype in CF mice [10]. This demonstrates the benefit of improving the hydration state of the intestinal lumen on the CF phenotype, and supports the rationale to explore therapies that activate fluid secretion mechanisms.

In this work we used the synthetic bioactive bicyclic fatty acid lubiprostone. Lubiprostone was approved by the FDA for treatment of chronic constipation in 2006. It is believed to have beneficial effects via enhanced fluid secretion and perhaps by increasing gut motility [14], although the latter effect is not firmly established as yet [15,16]. Lubiprostone increases fluid secretion from CF respiratory epithelial cells [2] and maybe also from airway submucosal glands [17]. Also, a recent case study reported improvement of constipation in adult CF patients [18]. The mechanism of action of lubiprostone is controversial, as discussed in a recent review [19]. Lubiprostone has been shown to activate the CLC2 (also known as CLCN2) Cl^- ^channel and stimulate apical fluid secretion that is independent of CFTR function [2,20-22]. However, lubiprostone can also activate the CFTR Cl^- ^channel [20,23,24]. There are also data suggesting that lubiprostone-induced chloride secretion is completely CFTR-dependent [23]. Despite these controversial issues, the encouraging results using lubiprostone in CF patients [18] and with CF cells and tissues [2,17], made it reasonable to test the hypothesis that lubiprostone would ameliorate the intestinal phenotype in the *Cftr *knockout mouse.

## Methods

### Animals

Mice with a targeted null mutation in the *Cftr *gene (*Cftr^tm1UNC^*) were originally obtained from Jackson Labs. They have been bred onto the C57BL/6J background until congenic [9]. *Cftr *heterozygous mice were bred to obtain *Cftr *null (CF) and wild type (WT) littermates. All mice, including WT and CF control and experimental mice, were fed a liquid diet (Peptamen, Nestle Nutrition, Florham Park, NJ, USA) which prevents lethal intestinal obstruction in CF mice. Administration of lubiprostone (Takeda Pharmaceutical North America, Deefield, IL, USA) was by a once daily gavage of 100 μl Peptamen containing a stable emulsion of lubiprostone. Preliminary experiments showed that a dose of 10 μg/kg-day was maximally effective (this dose maximally affected gastric emptying, data not shown). Mice were treated with lubiprostone for a period of 2 weeks before sacrifice. The effect of lubiprostone on body weight gain was assessed. To increase the statistical power of these comparisons, weights of CF mice were calculated as percent of WT mice of the same age, gender, and treatment group as previously described [10]. All animal work was approved by the Institutional Animal Use and Care Committe, of the Univeristy of Kansas Medical Center.

### Morphometry

The width of crypts stained with periodic acid-Shiff base, Alcian blue, pH 2.5 (PAS/AB) was used to estimate the amount of mucus accumulation as previously described [10]. Briefly, mice were fasted overnight with free access to water, and after sacrifice the small intestines were carefully resected keeping the luminal contents in place. The tissues were fixed overnight in Carnoy's solution to preserve mucus. The middle portion of the small intestine was used [10], and tissues were paraffin embedded and processed for PAS/AB staining (performed by Mass Histology, Worcester, MA). From each animal (at least 5 per group), 4 sections were imaged and crypt regions were recorded on a digital camera using a 20X objective on a Nikon microscope. Between 200-300 crypts per sample were imaged and measured using NIH Image J software as described [10]. For each group, the averages of the mean crypt width of each animal were determined and these averages (n ≥ 5 per group) used for statistical analysis.

### Estimation of bacterial load

The copy number of the bacterial 16*S *rRNA gene per intestine was used as an estimate of bacterial load in the small intestine as previously described [10]. Briefly, mice were fasted overnight with free access to water. The entire small intestine was resected and flushed with PBS containing the mucolytic reducing agent dithiothreitol (10 mM). The flushed material was centrifuged and the pellet was processed to extract bacterial DNA, using the QIAamp DNA Stool Mini Kit (Qiagen, Valencia, CA, USA) with minor modifications as previously reported [4]. The DNA was used to amplify the bacterial 16*S *rRNA gene with universal primers by quantitative PCR.

### Measurement of gastric emptying and small intestinal transit

Gastric emptying and small intestinal transit were measured as previously described [10]. Briefly, mice were fasted overnight and in the morning they were gavaged with the nondigestible, nonabsorbable tracer rhodamine dextran (70 kDa, Sigma). Twenty min later, mice were sacrificed and the distribution of fluorescence in the stomach and 10 equal segments of the small intestine was measured. There was no tracer in the cecum or colon in any of the mice at 20 min postgavage. Gastric emptying was calculated from the amount of tracer remaining in the stomach relative to the total fluorescence. Small intestinal transit was measured from the distribution of fluorescence in the small intestine (excluding that left in the stomach). For statistical analysis of small intestinal transit, the data were converted to geometric center of the fluorescence (GCF) = Σ (fraction per segment × segment number).

### Analysis of gene expression

Equal amounts of total RNA from individual samples were pooled from mice in each group and were used to interrogate the Affymetrix Mouse 430 2.0 GeneChip (Affymetrix, Santa Clara, CA, USA). We used 8 WT control, 7 WT + lubiprostone, 10 CF control, and 8 CF + lubiprostone RNA samples to create the pools for analysis (sample size n = 1 per group). Microarray data were analyzed using the GeneChip Operating System (GCOS) software (Affymetrix), using the WT control data as the baseline. The full microarray dataset is deposited in NCBI's Gene Expression Omnibus [25] and is accessible through GEO Series accession number GSE18327 http://www.ncbi.nlm.nih.gov/geo/query/acc.cgi?acc=GSE18327. Changes in expression of genes of interest from the microarrays were verified using quantitative RT-PCR. Briefly, total RNA was prepared from the entire small intestine by the Trizol method as previously described [4]. Real time qRT-PCR was performed with an iCycler instrument (Bio-Rad, Hercules, CA, USA) using a one-step RT-PCR kit (Qiagen). The mRNA for ribosomal protein L26 (*Rpl26*) was used as a housekeeping gene for normalization. Expression levels were calculated using the ΔΔC_t _method after correcting for differences in PCR efficiencies [26], and were expressed relative to WT control levels.

### Statistics

Data are presented as means ± SE. Significance was determined with Systat 11 software (San Jose, CA) by ANOVA, or Mann Whitney U test when data were not normally distributed. *P*-values < 0.05 were considered as significant.

## Results

### Body weight gain

**Figure 1:**
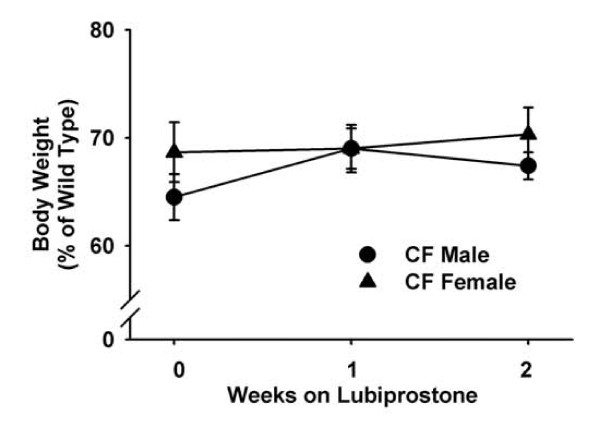
**Effect of lubiprostone treatment on body weight gain in CF male and female mice**. Body weights of lubiprostone treated CF mice are expressed as percent of WT mice of the same age and gender. Data are means ± SEM from lubiprostone treated mice (6 WT males, 6 WT females, 10 CF males, 10 CF females). There were no significant differences.

One of the most obvious effects of CF is poor body weight gain in early life. Although CF mice are pancreatic sufficient, in contrast to most CF patients, CF mice are about 70% the mass of WT mice from before weaning into early adulthood [27]. Lubiprostone was administered for 2 weeks and body weight gain was measured in WT and CF mice. Lubiprostone did not affect weight gain in WT mice as compared to control WT. Similarly, lubiprostone did not improve body weight gain in CF mice over the two week treatment period (Fig. 1).

### Mucus accumulation

**Figure 2:**
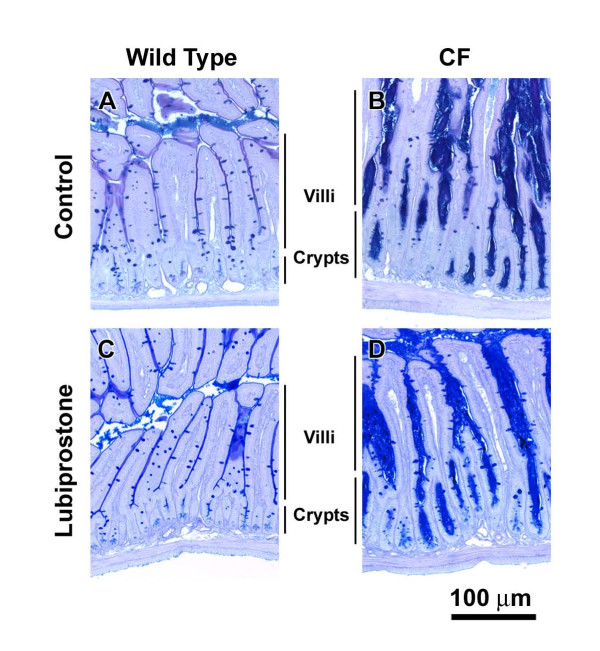
**Morphology of WT and CF small intestine without and with lubiprostone treatment**. Mice were treated with lubiprostone (10 μg/kg-day) for two weeks, as indicated. After an overnight fast, the small intestine was fixed in Carnoy's solution and processed for PAS/AB staining. The CF intestine has much greater mucus in the crypts as well as along the villus surfaces, as compared to WT tissue. There was not an obvious effect of lubiprostone on mucus in either WT or CF mice as compared to controls.

**Figure 3:**
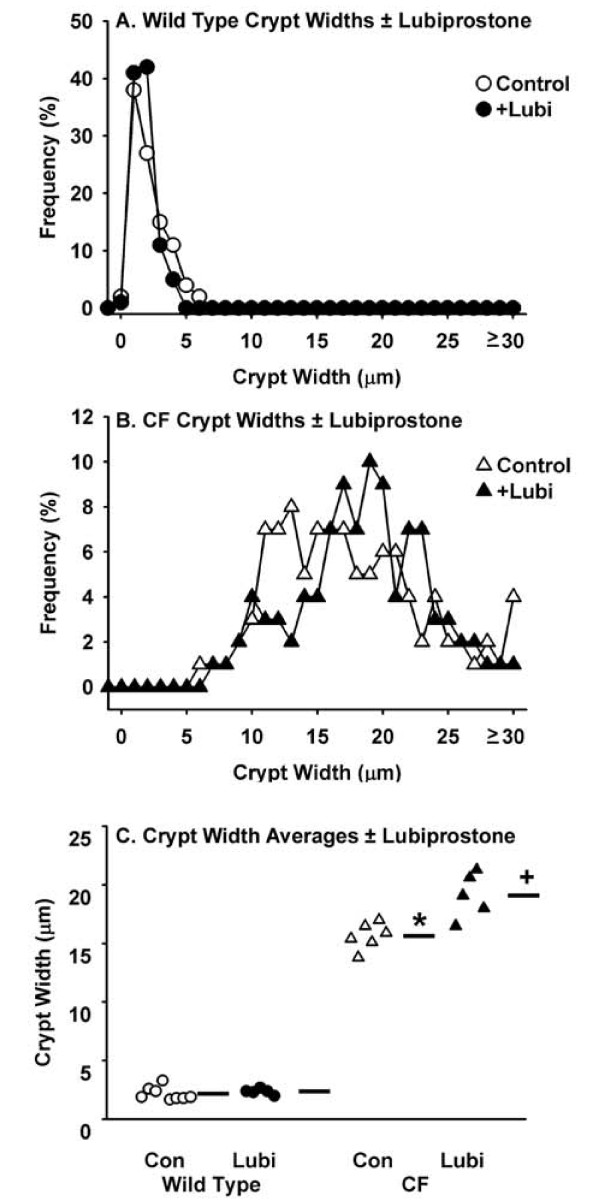
**Effect of lubiprostone treatment on mucus accumulation in the small intestine as measured by crypt lumen width in WT and CF mice**. Crypt widths of Carnoy's fixed, PAS/AB stained sections were used as an estimate of mucus accumulation. A. Frequency distribution of crypt widths in WT control and lubiprostone (+Lubi) treated mice. B. Frequency distribution of crypt widths in CF control and lubiprostone (+Lubi) treated mice. C. Summary of crypt widths in control (Con) and lubiprostone (Lubi) treated WT and CF mice. Horizontal bars represent the group means. (8 WT control, 5 WT + lubiprostone, 6 CF control, 5 CF + lubiprostone; *: *P *< 0.00001 CF control vs. WT control; + *P *= 0.00004 CF +Lubi vs. CF control)

A major phenotype in the CF intestine is accumulation of mucus (Fig. 2B), believed to be due to the dehydrated acidic luminal environment that occurs in the absence of normal CFTR function. Morphologically, there was not a noticeable effect of lubiprostone treatment on either WT (Fig. 2C) or CF (Fig. 2D) intestinal tissue. To be more rigorous, the effect of lubiprostone treatment on mucus accumulation was estimated from the width of small intestinal crypts, as previously described [5]. There was no effect of lubiprostone on crypt width in WT mice (Fig. 3A,C). Crypt width in control CF mice was ~700% that of control WT mice (Fig. 3). It was expected that lubiprostone would improve hydration and reduce mucus accumulation in the CF mouse. Unexpectedly, in lubiprostone treated CF mice the crypt the width was 22% greater than control CF mice (Fig. 3B,C).

### Bacterial load

**Figure 4:**
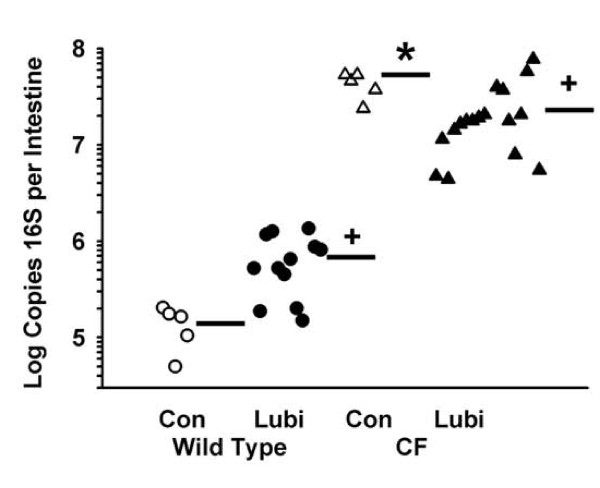
**Effect of lubiprostone on bacterial load in the small intestinal lumen of WT and CF mice**. After an overnight fast, the contents of the small intestine were flushed with a mucolytic agent, and the flushed material was processed to measure copies of the bacterial 16 S rRNA gene by quantitative PCR (see Materials and Methods). Horizontal bars represent the group means. [5 WT control (Con), 12 WT + lubiprostone (Lubi), 6 CF control, 18 CF + lubiprostone; *: *P *= 0.008 CF control vs. WT control; +: *P *< 0.010 +Lubi vs. control of same genotype]

We had hypothesized that lubiprostone would reduce mucus accumulation in the CF intestine. Since bacteria colonize and grow in the accumulated mucus of the CF intestine [4], we expected that lubiprostone would reduce the bacterial load in the CF intestine. Therefore, the effect of lubiprostone on bacterial load was examined. In WT mice treatment with lubiprostone increased bacterial load to almost 500% that of control WT mice (Fig. 4). As previously reported [4] the bacterial load in control CF mice was more than 10,000% that of control WT mice (Fig. 4). In lubiprostone treated CF mice there was a significant reduction in bacterial load by 60% as compared to control CF mice (Fig. 4).

### Gastrointestinal transit

**Figure 5:**
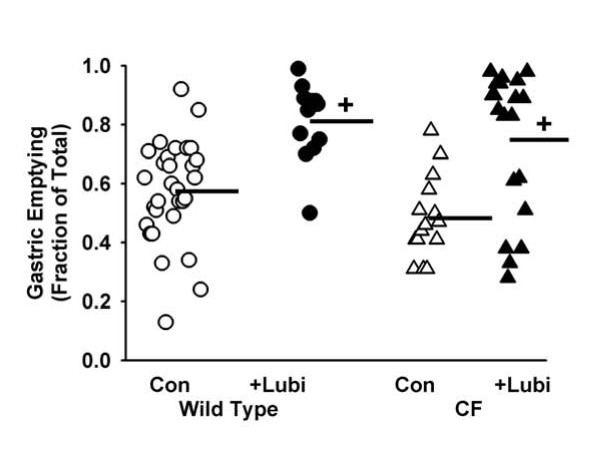
**Effect of lubiprostone on gastric emptying in WT and CF mice**. Mice were fasted overnight and in the morning gavaged with the nondigestible, nonabsorbable tracer rhodamine dextran (70 kDa). Twenty minutes later the mice were sacrificed and the amount of tracer remaining in the stomach relative to the gavaged amount was measured. Horizontal bars represent the group means. [30 WT control (Con), 12 WT + lubiprostone (Lubi), 15 CF control, 20 CF +Lubi; +: *P *< 0.001 +Lubi vs. control of same genotype]

The CF phenotype also includes effects on gastrointestinal motility [28]. Gastric emptying in CF mice is not significantly different from that in WT at 20 min postgavage [28], but it is less at 90 min postgavage [29]. In the current work, we confirmed that at 20 min postgavage there is not a significant difference in gastric emptying comparing control CF to WT (Fig. 5). In lubiprostone treated mice, gastric emptying was significantly greater in both WT and CF mice as compared to the respective control animals (Fig. 5).

**Figure 6:**
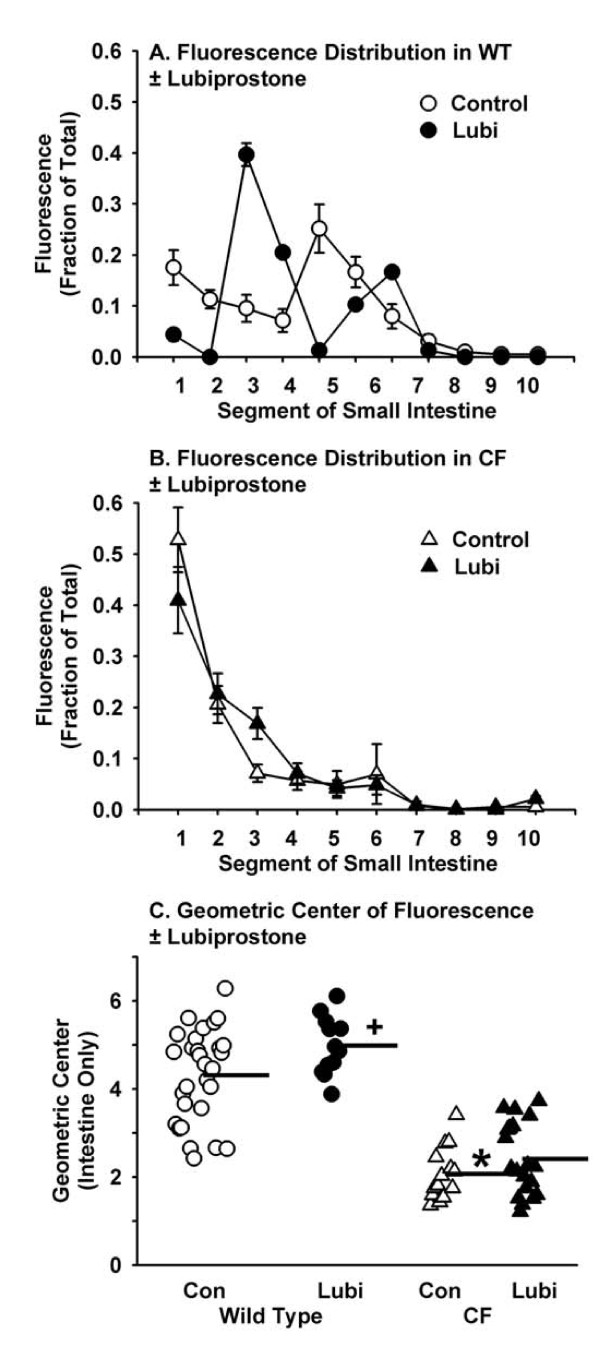
**Effect of lubiprostone on small intestinal transit in WT and CF mice**. Mice were fasted overnight and in the morning gavaged with the nondigestible, nonabsorbable tracer rhodamine dextran (70 kDa). Twenty minutes later the mice were sacrificed and the distribution of tracer along the small intestine was measured. A. Distribution of rhodamine along the intestine of WT control and lubiprostone (+Lubi) treated mice. B. Distribution of rhodamine along the intestine of CF mice control and lubiprostone treated mice. C. Geometric center of fluorescence in control (Con) WT and CF mice and lubiprostone (Lubi) treated WT and CF mice. Horizontal bars represent the group means. [30 WT control, 12 WT + lubiprostone, 15 CF control, 20 CF + lubiprostone; *: *P *< 0.00001 CF control vs. WT control; + *P *= 0.03 WT +Lubi vs. WT control]

CF mice have strongly impaired small intestinal transit as compared to WT [28] which was confirmed here (Fig. 6A, B). Small intestinal transit was slightly greater in lubiprostone treated WT mice but not in treated CF mice (Fig. 6A, B). The transit data were statistically analyzed by transforming them to the geometric center of fluorescence (GCF) values. The GCF in lubiprostone treated WT mice was significantly greater as compared to control WT (Fig. 6C). There was not a significant difference in intestinal transit comparing lubiprostone treated CF to control CF mice.

### Microarray and qRT-PCR analysis of gene expression

There are numerous changes in gene expression in the CF mouse small intestine as revealed by analysis of global expression levels using the Affymetrix U74Av2 GeneChip [9]. Therefore, it was of interest to see how lubiprostone treatment affected gene expression in WT and CF mice. Because we had previously performed this type of analysis in control WT and CF mice, in the current work we used total RNA samples pooled by group to investigate global gene expression effects of lubiprostone, using the newer Mouse 430 2.0 GeneChip (see Methods). The major changes in gene expression in the CF intestine reported previously were replicated in the new analysis (both complete datasets are deposited at Genome Expression Omnibus website, GEO Series accession numbers GSE765 and GSE18327, respectively, at http://www.ncbi.nlm.nih.gov/geo/query/acc.cgi?acc=GSE765 and http://www.ncbi.nlm.nih.gov/geo/query/acc.cgi?acc=GSE18327. We used an arbitrary cutoff of 3-fold to select genes whose expression was changed by lubiprostone treatment. We chose several of the genes indicated by the arrays for verification by quantitative RT-PCR.

There were 18 genes on the arrays whose expression was ≥ 3-fold greater in the CF control sample as compared to WT control, that were also downregulated ≥ 3-fold by lubiprostone treatment of CF mice (Table 1). Ten of these genes are associated with immunity, eight of which are involved with innate immunity including five markers of mast cells. Another four genes are associated with growth or differentiation, three of which are members of the *gasdermin *gene family. The remaining three genes are involved in various functions that do not fall into obvious functional groups.

**Table 1:** Genes upregulated in the control CF intestine as compared to control WT, and downregulated by lubiprostone treatment of CF mice.

CF	WT+L	CF+L	Symbol	Name	Function/Process
1176	1.52	365	*Retnlb*	resistin like beta	Innate immunity

112	3.11	4.5	*Chi3l4*	chitinase 3-like 4	Innate immunity

51	0.52	8.7	*Pla2g4c*	phospholipase A2, group IVC (cytosolic, calcium- independent)	Inflammation, eicosanoid metabolism

42	0.76	3.4	*Mcpt1*	mast cell protease 1	Innate immunity, mast cell

23	1.32	2.8	*Spna1*	spectrin alpha 1	Erythrocyte cytoskeleton

18	0.23	1.3	*Gsdmc2*	gasdermin C2	Growth, differentiation, apoptosis

16	0.76	1.4	*Mcpt2*	mast cell protease 2	Innate immunity, mast cell marker

14	1.15	2.1	*Mcpt9*	mast cell protease 9	Innate immunity, mast cell

12	1.32	3.7	*B3galt5*	UDP-Gal:betaGlcNAc beta 1,3-galactosyltransferase, polypeptide 5	Sialyl Lewis-a synthesis

11	4.92	2.1	*Ube2l6*	ubiquitin-conjugating enzyme E2L 6	Innate immunity, ISG15- ubiquitination

8.6	1.00	1.2	*Capn13*	calpain 13	Growth, differentiation, apoptosis

8.0	0.23	1.4	*Gsdmc4*	gasdermin C4	Growth, differentiation, apoptosis

7.0	0.27	1.3	*Gsdmc*	gasdermin C	Growth, differentiation, apoptosis

4.9	0.44	1.4	*Cpa3*	carboxypeptidase A3, mast cell	Innate immunity, mast cell marker

4.0	0.76	0.2	*Fcer1a*	Fc receptor, IgE, high affinity I, alpha polypeptide	Innate immunity, mast cell marker

3.7	1.15	1.4	*Egln3*	EGL nine homolog 3 (C. elegans)	Prolyl hydroxylation of HIF

3.5	1.62	1	*(none)*	single chain antibody ScFv	Immunoglobulin heavy chain, immunity

3.2	0.56	0.5	*Scd2*	stearoyl-Coenzyme A desaturase 2	Unsaturated fatty acid synthesis

Genes whose expression level in the CF control intestine were ≥ 3-fold greater than WT control and were downregulated ≥ 3-fold by lubiprostone in CF mice. Expression values are data from the GCOS analysis relative to WT control levels. When there were multiple probe sets for a particular gene the values were averaged. Gene names are according to the Mouse Genome Informatics website http://www.informatics.jax.org/. +L = lubiprostone treated. Equal amounts of total RNA were pooled from 7-10 mice in each group and were used to interrogate the Affymetrix Mouse 430 2.0 GeneChip (n = 1 per group). The full microarray dataset is deposited in NCBI's Gene Expression Omnibus and is accessible through GEO Series accession number GSE18327 http://www.ncbi.nlm.nih.gov/geo/query/acc.cgi?acc=GSE18327.

**Figure 7:**
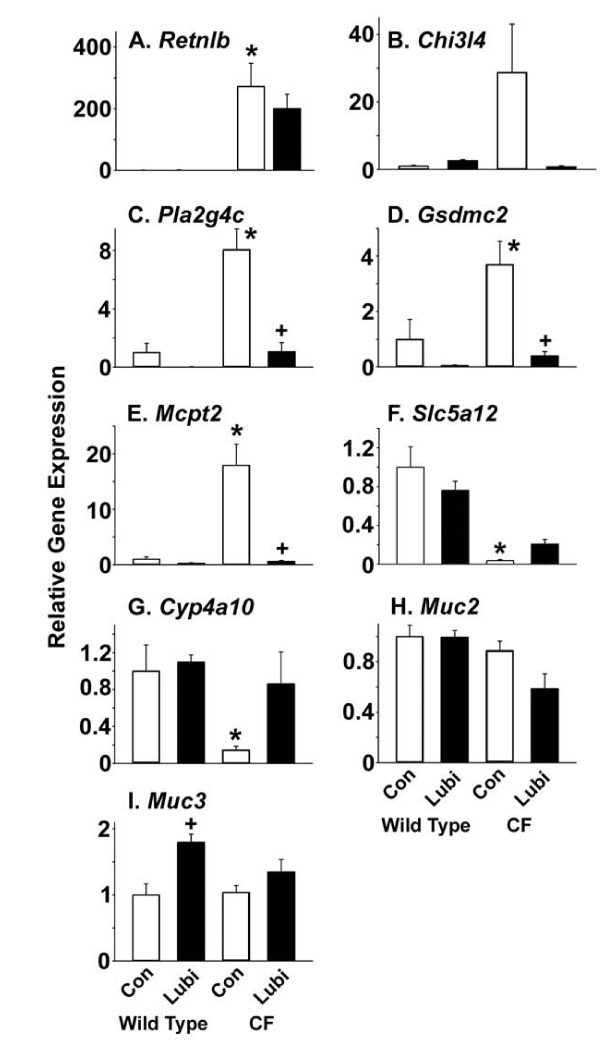
**Verification of microarray results for selected genes by qRT-PCR**. (A) *Retnlb*, resistin-like β, also known as Fizz2; (B) *Chi3l4*, chitinase 3-like 4, also known as Ym2; (C) *Pla2g4c*, phospholipase A2, group 4c; (D) *Gsdmc2*, gasdermin c2; (E) *Mcpt2*, mast cell protease 2; (F) *Slc5a12*, sodium-monocarboxylate transporter; (G) *Cyp4a10*, cytochrome P450 4a10; (H) *HemT1*, hematopoietic cell transcript 1; (I) *Muc2*, mucin 2; (J) *Muc3*, mucin 3. All data are normalized to expression of ribosomal protein 26 (*Rpl26*) and are relative to WT control expression levels. Data are means ± SEM; *: *P <*0.05 comparing CF control to WT control; +: *P *< 0.05 +Lubi compared to control of same genotype. (n = 8 WT controls, 6 WT lubiprostone treated, 8 CF controls, 6 lubiprostone treated CF samples)

By qRT-PCR we examined 5 of the genes that were indicated on the arrays as being upregulated in CF compared to WT, and downregulated in CF treated with lubiprostone. As previously reported [9], resistin-like β (*Retnlb*) was very highly increased in the CF intestine (Fig. 7A). Whereas the arrays indicated a 3-fold decrease in *Retnlb *expression in the lubiprostone treated CF intestine, there was not a significant decrease as measured by qRT-PCR (Fig. 7A). The arrays showed a large upregulation of chitinase 3-like 4 (*Chi3l4*, also known as *Ym2*) (Table 1). By qRT-PCR there were similar changes in *Chi3l4 *expression but because of the high variability in the CF control group, the differences were not statistically significant (Fig. 7B). We were able to confirm by qRT-PCR that phospholipase A2 group 4c (*Pla2g4c*), gasdermin c2 (*Gsdmc2 *also known as *MLZE*, melanoma-derived leucine zipper-containing extranuclear factor), and mast cell protease 2 (*Mcpt2*) (Table 1), were all upregulated in CF controls and downregulated to WT control levels in lubiprostone treated CF mice (Fig. 7C-E). In addition to *Mcpt2*, several other mast cell markers on the arrays were strongly upregulated in CF controls and decreased 3-20 fold in lubiprostone treated CF mice (Table 1).

There were 11 genes whose expression was ≥ 3-fold lesser in the CF control sample as compared to WT controls, and whose expression was upregulated ≥ 3-fold by lubiprostone treatment of CF mice (Table 2). Four of these genes are involved in lipid metabolism, including production of eicosanoids. Another three genes are involved in glucose metabolism (Table 2). And, another three are involved in solute transport (Table 2). By qRT-PCR we verified these changes for *Slc5a12 *(sodium monocarboxylate transporter) and *Cyp4a10 *(cytochrome P450 4a10, which converts arachidonic acid to 20-HETE) (Fig. 7F and 7G, respectively).

**Table 2:** Genes downregulated in the control CF intestine as compared to control WT, and upregulated by lubiprostone treatment of CF mice.

CF	WT+L	CF+L	Symbol	Name	Function/Process
0.01	0.4	0.11	*Slc5a12*	solute carrier family 5 (sodium/glucose cotransporter), member 12	Na-monocarboxylate transporter

0.05	0.4	0.33	*Cyp4a10*	cytochrome P450, family 4, subfamily a, polypeptide 10	20-HETE synthesis

0.08	0.9	0.46	*Pck1*	phosphoenolpyruvate carboxykinase 1, cytosolic	Glucose metabolism

0.12	0.6	1.1	*Pdk4*	pyruvate dehydrogenase kinase, isoenzyme 4	Glucose metabolism

0.15	1.3	0.53	*Fbp1*	fructose bisphosphatase 1	Glucose metabolism

0.21	1.2	2.14	*Hmgcs2*	3-hydroxy-3-methylglutaryl-Coenzyme A synthase 2	Ketogenesis

0.21	1.1	1.74	*Acot2*	acyl-CoA thioesterase 2	Fatty acid metabolism

0.25	1.1	0.93	*Slc13a2*	solute carrier family 13 (sodium-dependent dicarboxylate transporter), member 2	Na-dicarboxylate transporter

0.25	1.1	1.33	*Sgk1*	serum/glucocorticoid regulated kinase 1	Regulation of electrolyte transport

0.33	1.6	1.32	*Akr1b7*	aldo-keto reductase family 1, member B7	PGF_2_a synthesis

0.33	1.1	2.15	*Angptl4*	angiopoietin-like 4	Lipid metabolism

Genes whose expression level in the CF control intestine were ≥ 3-fold less than WT control and were upregulated ≥ 3-fold by lubiprostone in CF mice. Expression values are data from the GCOS analysis relative to WT control levels. Gene names are according to the Mouse Genome Informatics website http://www.informatics.jax.org/. +L = lubiprostone treated. Equal amounts of total RNA were pooled from 7-10 mice in each group and were used to interrogate the Affymetrix Mouse 430 2.0 GeneChip (n = 1 per group). The full microarray dataset is deposited in NCBI's Gene Expression Omnibus and is accessible through GEO Series accession number GSE18327 http://www.ncbi.nlm.nih.gov/geo/query/acc.cgi?acc=GSE18327.

On the arrays, there were 4 genes upregulated ≥3-fold in lubiprostone treated WT mice as compared to WT control, and 40 genes upregulated ≥3-fold in lubiprostone treated CF mice as compared to CF control (see http://www.ncbi.nlm.nih.gov/geo/query/acc.cgi?acc=GSE18327). None of these genes was upregulated in common in both WT and CF lubiprostone treated mice. Also, there were 24 genes whose expression was downregulated ≥ 3-fold in lubiprostone treated WT mice as compared to WT control, and 32 genes downregulated ≥ 3-fold in lubiprostone treated CF mice as compared to CF control (see http://www.ncbi.nlm.nih.gov/geo/query/acc.cgi?acc=GSE18327). None of these genes was downregulated in common in both WT and CF lubiprostone treated mice.

Since mucus accumulation in CF crypts appeared to be increased by lubiprostone treatment, we also measured expression levels of the two major mucin genes expressed in the small intestine, *Muc2 *and *Muc3*. There were no significant differences in *Muc2 *expression levels comparing any of the four groups of mice (Fig. 7H). Expression of *Muc3 *was modestly but significantly increased in lubiprostone treated WT mice, but there was not a significant difference in lubiprostone treated CF mice (Fig. 7I).

## Discussion

Cystic fibrosis is caused by loss of function of the cAMP regulated anion channel CFTR. Therefore, we hypothesized that lubiprostone, which has been reported to activate the non-CFTR Cl^- ^channel CLC2, would improve the CF intestinal phenotype in *Cftr *knockout mice. Administration of lubiprostone improved aspects of the CF phenotype, notably small intestinal bacterial overgrowth and the innate immune response, but did not affect others, including small intestinal transit and body weight gain.

### Effect on mucus accumulation

It was hypothesized that if lubiprostone improved the hydration of mucus this would allow more normal mucus turnover in the CF intestine. We estimated mucus accumulation morphometrically by the width of PAS/AB stained crypts, and unexpectedly, the crypt width was significantly increased in lubiprostone treated CF mice as compared to control CF mice. One possible explanation is that fluid transport stimulated by lubiprostone increases the hydration of released mucins (the highly glycosylated proteins that form mucus) causing the mucus to swell more, and thus widening the crypt lumen. If this explanation is correct, it would suggest that the amount of fluid secretion induced by lubiprostone was not sufficient to flush out the *cul-de-sac *that is the intestinal crypt, which in CF has excessive mucin secretion.

Another possibility is that lubiprostone-induced fluid secretion enhances mucin release from intestinal goblet cells. It has been demonstrated that mucin release from a goblet cell-like cell line is accompanied by Cl^- ^dependent fluid secretion, suggesting a functional coupling between these two processes [30]. Also, it was shown that CFTR function supports efficient release of soluble mucin from the mouse small intestine [31] and CFTR has been implicated in mucin release from a tracheal gland cell line [32]. Lubiprostone has been shown to stimulate duodenal bicarbonate (HCO_3_^-^) secretion [20] and HCO_3_^- ^ion supports efficient intestinal mucin release [31].

An alternate possibility is that lubiprostone acts as a secretagogue that stimulates an intracellular messenger pathway leading to mucin exocytosis from goblet cells. It is known that PGE_2 _is a potent mucin secretagogue [31]. There is also evidence that lubiprostone can activate PGE_2 _receptors [23,33], but this has not been universally confirmed [24,34]. If lubiprostone stimulates mucin release, the fact that crypt width was not increased in lubiprostone-treated WT mice would suggest that there is sufficient fluid secretion in WT mice to prevent mucus accumulation in the crypt lumen as occurs in CF. Thus, lubiprostone may enhance mucin release through its effects on fluid secretion, or more directly through activation of mucin granule exocytosis via prostaglandin receptor activation.

### Effect on bacterial load

It was predicted that lubiprostone would reduce bacterial overgrowth of the CF small intestine, and there was a significant 60% reduction in bacterial load in lubiprostone treated CF mice. An obvious mechanism is that enhanced fluid secretion improves solubility and turnover of luminal mucus that bacteria colonize. It was unexpected that lubiprostone treatment would affect bacterial load in WT mice, but we observed increased bacterial load of ~500%. This result does not appear compatible with an effect of lubiprostone on fluid secretion; increased fluid secretion is not expected to increase the ability of bacteria to colonize and grow in the WT mouse intestine. However, if lubiprostone also stimulates mucin secretion from goblet cells, it could be that more mucus in the intestinal lumen is available for bacterial colonization and growth in WT mice. One would then have to propose that greater mucus release in the CF intestine, in combination with enhanced anion and fluid secretion, is sufficient to increase mucus clearance and thereby reduce the bacterial load in lubiprostone treated CF mice. Because of the complex behavior of mucus, it is difficult to know if this explanation can account for the opposite effects of lubiprostone on bacterial load in the WT vs. CF small intestine.

### Effects on the CF intestine innate immune response

Treatment of CF mice with lubiprostone decreased expression of several innate immunity genes. Included in these are markers of mast cells which are dramatically increased in the control CF intestine and whose expression was reduced to WT levels in lubiprostone treated CF mice. This indicates that the dramatic influx of mast cells into the CF mouse small intestine [9] is reversed by lubiprostone treatment. Another gene involved in inflammation, *Pla2g4c*, calcium-independent phospholipase A2, group 4c (also known as PLA2γ) was strongly upregulated in the CF intestine and downregulated to WT control levels by lubiprostone treatment. This enzyme was shown to be induced in jejunal epithelium by nematode infection of the small intestine [35]. Our results indicate that *Pla2g4c *expression is also increased by bacterial overgrowth which occurs in the CF intestine and lubiprostone reverses its upregulation.

It remains to be determined how lubiprostone decreased the innate immune response in CF mice. One possibility is that, since the bacterial load was significantly decreased by lubiprostone treatment, this may have reduced the immune response. However, in a previous study we showed that oral administration of the mucolytic *N*-acetylcysteine reduced bacterial load to the same extent as in lubiprostone treated CF mice, but there was no improvement in the innate immune response gene expression levels with *N*-acetylcysteine [10]. Another possibility is that lubiprostone activates a prostaglandin receptor and has anti-inflammatory or some other protective effect. Prostaglandins, to which lubiprostone is structurally related, are gastroprotective in large part due to their stimulation of bicarbonate and mucus secretion. Perhaps lubiprostone acts in a similar fashion to improve the innate response of the CF intestine. Lubiprostone has been reported to stimulate bicarbonate secretion which was suggested to be through activation of the EP4 PGE_2 _receptor [20] and activation of Cl- secretion was blocked by an EP4 receptor antagonist [23]. However, other work has shown that lubiprostone does not have appreciable affinity for the EP4 receptor [24,33], so the mechanism by which lubiprostone stimulates bicarbonate secretion remains uncertain [19].

A family of genes whose expression was upregulated in the CF intestine and downregulated by lubiprostone treatment are the gasdermins. The gasdermins have been shown in cell culture to be anti-apoptotic and are thought to be involved in regulation of growth and differentiation [36]. It is interesting to note that the epithelium of the CF mouse small intestine is hypertrophied and both crypts and villi are larger than in WT mice [10,37], which is consistent with the role of gasdermins. However, even though expression of gasdermins was downregulated in lubiprostone-treated CF mice, the epithelium was still hypertrophied (Fig. 2D). It may be that the two week treatment period was not sufficient to reverse the hypertrophic state of the epithelium, or that other factors are of greater importance.

### Effects on GI motility

We observed two effects of lubiprostone on GI motility. First, lubiprostone increased gastric emptying in both WT and CF mice. CLC2 is present in the stomach and was suggested to be the Cl^- ^channel required for gastric acid secretion [38]. Lubiprostone-induced fluid secretion in the stomach may increase the rate of emptying of the water soluble tracer used (rhodamine-dextran), which would explain the stimulatory effects of lubiprostone on gastric emptying we observed. In contrast, in a human study, lubiprostone was shown to increase fasting gastric volume and inhibit emptying of a solid nutrient test meal [14]. A likely explanation for these different results is that we used non-nutritive tracer and vehicle in our work, and this is expected to measure activity of the interdigestive migrating motor complex; on the other hand, the human study used a solid nutrient test meal, which in itself will affect motility programs, and this model will measure postprandial motility.

The mechanism of action of lubiprostone is the subject of debate [19]. Early work using cell transfection studies showed that Cl^- ^transport stimulated by lubiprostone was mediated by the CLC2 gene product [22]. However, at drug concentrations >100 nM, the CFTR Cl^- ^channel is also activated [39]. A recent study using guinea pig ileum concluded that Cl^- ^secretory effects of lubiprostone were not mediated by CFTR, but were consistent with activation of CLC2 [24]. However, in another recent study using mice, various cell lines, and human control and CF patient intestinal tissues, it was concluded that the action of lubiprostone required CFTR [23]. Whether these disparate results are due to species or methodological differences is unclear at this point. Since we used *Cftr *null mice, the effects of lubiprostone in our experiments cannot be through the CFTR channel. If the Cl^- ^secretory response to lubiprostone in the mouse small intestine is CFTR-dependent as recently reported [23], our results would indicate that lubiprostone's effects in *Cftr *null mice are through another pathway, such as a prostaglandin receptor. Since PGE_2 _can stimulate murine duodenal HCO_3_^- ^ion secretion (via the SLC26A6 transporter but not through CFTR [40]), it may be that lubiprostone enhances HCO_3_^- ^secretion, which is expected to improve mucus solubility [31]. Despite the uncertainties regarding lubiprostone's mechanism of action, there were significant changes in lubiprostone treated CF mice which demonstrate that lubiprostone has CFTR independent effects in the mouse small intestine that are beneficial to the CF phenotype.

## Conclusions

Treatment of CF mice with lubiprostone ameliorates the CF intestinal phenotype. There was a significant decrease in bacterial overgrowth and normalized expression levels of several innate response genes, especially for mast cell markers. Because there are significant differences between mice and humans, it will be important to further test the potential intestinal benefits of lubiprostone in CF patients. It is an open question whether the beneficial effects of lubiprostone in the CF mouse are due to stimulation of Cl^- ^and fluid secretion. There is also the possibility that lubiprostone activates prostaglandin receptors, perhaps stimulating mucin and HCO_3_^- ^secretion, and having additional anti-inflammatory effects mediated through such receptors. If lubiprostone enhances mucin and HCO_3_^- ^release this drug also may be beneficial for various GI conditions where bolstering production of the protective mucus layer is desirable, such as gastric ulcer and enteric infection.

## Competing interests

This work was funded by grant 07-010 from Takeda Pharmaceuticals North America, Inc. Takeda had no input into the design, execution, or data analysis. Takeda made minor editorial comments on the manuscript. Takeda Pharmaceuticals is distributing the substance lubiprostone investigated in this study.

## Authors' contributions

RCD directed the work, helped analyze the data, and wrote the manuscript. RM performed some of the qRT-PCR determinations and maintained the mouse colony. ER performed and analyzed the morphometric study. All authors assisted in editing of the manuscript.

## Pre-publication history

The pre-publication history for this paper can be accessed here:

http://www.biomedcentral.com/1471-230X/10/107/prepub
